# Risks of Using Antifouling Biocides in Aquaculture

**DOI:** 10.3390/ijms13021541

**Published:** 2012-02-02

**Authors:** Francisco Antonio Guardiola, Alberto Cuesta, José Meseguer, Maria Angeles Esteban

**Affiliations:** Department of Cell Biology and Histology, Faculty of Biology, University of Murcia, Murcia 30100, Spain; E-Mails: faga1@um.es (F.A.G.); alcuesta@um.es (A.C.); meseguer@um.es (J.M.)

**Keywords:** biocides, antifouling, environment, aquaculture, resistance, antibiotics

## Abstract

Biocides are chemical substances that can deter or kill the microorganisms responsible for biofouling. The rapid expansion of the aquaculture industry is having a significant impact on the marine ecosystems. As the industry expands, it requires the use of more drugs, disinfectants and antifoulant compounds (biocides) to eliminate the microorganisms in the aquaculture facilities. The use of biocides in the aquatic environment, however, has proved to be harmful as it has toxic effects on the marine environment. Organic booster biocides were recently introduced as alternatives to the organotin compounds found in antifouling products after restrictions were imposed on the use of tributyltin (TBT). The replacement products are generally based on copper metal oxides and organic biocides. The biocides that are most commonly used in antifouling paints include chlorothalonil, dichlofluanid, DCOIT (4,5-dichloro-2-n-octyl-4-isothiazolin-3-one, Sea-nine 211^®^), Diuron, Irgarol 1051, TCMS pyridine (2,3,3,6-tetrachloro-4-methylsulfonyl pyridine), zinc pyrithione and Zineb. There are two types of risks associated with the use of biocides in aquaculture: (i) predators and humans may ingest the fish and shellfish that have accumulated in these contaminants and (ii) the development of antibiotic resistance in bacteria. This paper provides an overview of the effects of antifouling (AF) biocides on aquatic organisms. It also provides some insights into the effects and risks of these compounds on non-target organisms.

## 1. Introduction

**Figure 1:**
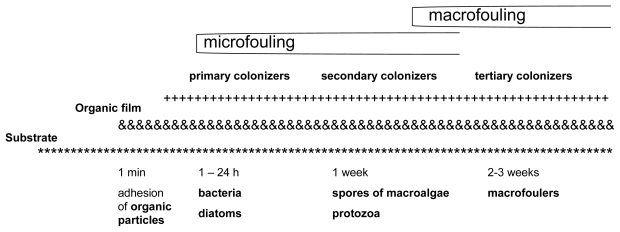
Temporal organization of biofouling (modified from [5]).

According to the Biocides Directive (98/8/EC) [1], biocides are active substances or preparations that are intended to destroy, deter, render harmless and exercise control or prevent the action of any other harmful organism through chemical or biological means. Biocides are classified into 23 different product types, each of which is comprised of multiple subgroups. Biocides are used because of their potential to destroy a wide range of organisms and for their relatively easy applicability to vessels [2] and aquaculture systems. The settlement of microorganisms, plants and animals is a natural phenomenon that occurs continuously and vigorously on immersed surfaces. This process is called biofouling [3] (Figure 1). Biofouling is a problem for any structure placed in the aquatic environment. It can be controlled through the use of both chemical biocides and non-biocidal technologies [4].

Aquaculture in general, and the fish farming industry in particular, suffer significantly from the effects of biofouling [6]. The aquaculture industry makes periodic discharges of wastes from farm activities. These waste products include detergents, effluent from net washing, antifoulants, heavy metals and even chemicals, such as drugs [7]. The chemicals are essential for aquaculture as they help increase and control the production of seeds in hatcheries, increase feeding efficiency, improve survival rates, control pathogens and diseases and reduce transport stress [8,9].

Nevertheless, despite the beneficial effects of the chemicals to aquaculture, they may also cause potential harm to aquatic organisms [10–12] and even to humans. The chemicals may be ingested by farmed fish and shellfish, which are, in turn, consumed by humans. Ingestion of the contaminated fish and shellfish can pose a great risk to human health [9,13]. The conditions and locations of the aquaculture farms play a significant role on the spread of these chemicals and heavy metals into the environment [14].

Marine pollution caused by the chemicals utilised in aquaculture activities, however, is not yet well documented. In addition, available information indicates low concentrations (low ng L^−1^ level) [15] of these compounds in the environment. This is due to factors, such as the complexity of the matrix, the high dilution factor, and degradation phenomena [9,16]. Nevertheless, the health risk in animals and humans may increase when bacterial resistance to antibiotics and heavy metals caused by the use of biocides occurs [17–19]. The aim of this study is to review the main effects and risks of using antifouling biocides in aquaculture on aquatic systems, shellfish, fish and humans.

## 2. Present Situation of Biocide Use in Aquaculture

Aquaculture is the farming of aquatic organisms, including fish, molluscs, crustaceans and aquatic plants, using techniques designed to increase the production and productivity of these organisms beyond the natural capacity of the environment [9]. Since wild fish stocks are reaching the limits of exploitation, we have to rely to a far greater extent on products produced from aquaculture [20]. However, the practice of aquaculture has become so widespread that it has begun to have significant impact on the environment and on natural resources. A number of concerns have been expressed by both environmental activists and scientists regarding this practice [21–25].

With the rapid expansion of the aquaculture industry and with the tightening of the legislation on the use of antifouling (AF) biocides, the problems of aquaculture biofouling have increased [5,6,26,27]. The herbicides or fungicides currently used in aquaculture were originally developed for use in agriculture or as additives for boat anti-fouling paints. As such, the published data regarding their occurrence in marine waters are mainly related to such activities [15,28,29]. Accordingly, many studies have investigated and demonstrated the presence of pesticides and biocides in surface waters [30–34].

With the gradual elimination of triorganotin-based formulations (e.g., tributyltin (TBT)), copper has become the principal biocidal component of most AF paints. It usually comes in the form of copper oxide (Cu_2_O) [5]. Inorganic zinc is often used in combination with copper to increase the overall toxicity of the formulation or to facilitate the leaching process [35]. Organic booster biocides, such as Irgarol 1051^®^, Sea Nine 211^®^, dichlofluanid, chlorothalonil, zinc pyrithione, and Zineb are also added to the paint to enhance its effectiveness [36]. The main AFs used in aquaculture and their effect are shown in Table 1. Nevertheless, these alternatives to TBT are also toxic and their contamination of the aquatic environment has been a topic of increasing importance in recent years [29]. Several studies have evaluated the toxicity of booster biocides on non-target species and have found most of them to be growth inhibitors for freshwater and marine autotrophs [37], influencing key species, such as sea grasses [38] and corals [39]. Therefore, there is increasing interest in the impact of these compounds on the aquatic ecosystems [40].

In the aquatic environment, fishes have been found appropriate to be used as a model for the immunotoxicity testing because they are representatives of aquatic organisms and, therefore, bioindicators of aquatic animal health. As vertebrates that have immune systems strikingly similar to those of mammals, they can also be used to identify potential threats to terrestrial wildlife and humans [41,42]. The risk to predators and humans through the consumption of fish is very low, especially for humans, since the latter are less exposed to the dangers of contamination due to the fact that fish constitutes only a small part of their diet [9]. However, the risk may be increased by mechanisms of resistance.

## 3. The Main Type of Antifouling Used in Aquaculture and Its Effect on Aquatic Organisms

### 3.1. Chorothalonil

Chlorothalonil (2,4,5,6-tetrachloroisophthalonitrile) is a pesticide used widely in agriculture, silviculture and urban settings. This pesticide can enter surface waters through rainfall runoff, spray drift or atmospheric deposition, subsequently impacting aquatic biota [77]. It is used as a booster biocide in marine paints as one of the chemicals replacing the widely banned organotin fungicides, such as tributyltin, resulting in greater potential for chlorothalonil contamination of marine waters and sediments [78,79]. Chlorothalonil is a broad-spectrum fungicide with a K^ow^ of 2.64–4.28 and a water solubility of 0.9 mg L^−1^ [80].

Chlorothalonil can be acutely toxic (50% lethal concentration, LC_50_) to fish following 96 h exposures ranging from 8.2 to 76 μg L^−1^, depending on the species and the exposure conditions [48,51]. Chlorothalonil can accumulate in the tissue of fish. Bioaccumulation factors have been reported to be 18 for willow shiner (*Gnathopogon caerulescens*) and 25 for carp (*Cyprinus carpio*) following sublethal exposures (1.1–1.4 μg L^−1^) [81]. It has been suggested that leukocytes may be a potential target of toxicity because significant decreases in leukocyte values were found in the Australian freshwater fish *Pseudaphritis urvulii,* which was exposed for 10 d to 4.4 μg L^−1^ chlorothalonil [51]. *In vitro* studies have demonstrated that the exposure of fish (*Morone saxatilus*) macrophages and oyster hemocytes to chlorothalonil (10 ± 500 μg L^−1^) suppressed immunostimulated ROS (reactive oxygen species) and baseline NADPH (nicotinamide adenine dinucleotide phosphate) concentration but did not inhibit phagocytosis [82,83]. There are numerous toxicity studies for chlorothalonil on marine animals, such as crustaceans [43–46], molluscs [44–48], tunicates [47] and teleosts [44–46,49–51].

### 3.2. Copper Oxide

Copper is an essential metal. However, although it is an effective biocide, it may also affect non-target organisms and cause environmental concerns [84]. The toxicity of copper in water is greatly affected by the chemical form or speciation of the copper and to what degree it is bound to various ligands that may be in the water, making the copper unavailable to organisms [85]. The speciation is essential for understanding the copper’s bioavailability and subsequent toxicity to aquatic organisms [4]. Copper oxide leaches from the boat surfaces and enters the water as a free copper ion (Cu^+^ ), which is immediately oxidised to Cu^2+^ and forms complexes with inorganic and organic ligands [4].

Copper is a trace element needed at miniscule levels for the proper functioning of all organisms [84]. However, it can be toxic at higher concentrations [85]. Copper is generally toxic to aquatic organisms, with a lethal concentration 50 (LC_50_) value varying from 5 to 100,000 μg L^−1^ [86,87]. However, organisms have different mechanisms by which they cope with and process copper [84]. Generally, copper is actively regulated in fish, decapod crustaceans and algae. It is stored in bivalves, barnacles and aquatic insects [84,88].

The bioavailability, biodistribution to various parts of the organism and bioaccumulation of copper are dramatically influenced by water chemistry. Therefore, water pH, hardness, organic content and salinity play important roles in copper-induced toxicity [84,85]. Thus, increased pH accentuates copper toxicity because of the reduced competition between copper and hydrogen ions at the cell surface [84,89]. In a similar manner, cations that are involved in water hardness also compete with Cu^2+^ for biological binding sites [84,90].

Copper bound to organic matter is widely thought to be non-bioavailable and, therefore, non-toxic [4,91,92]. Dissolved organic carbon (DOC) content is among the most important factors in reducing copper toxicity in both fresh- and salt-water species [84]. DOC forms organic complexes with copper, thereby reducing copper’s bioavailability [84]. The effects of DOC on reducing the toxicity of copper have been reported in fish [93,94], bivalves [92], echinoderms [95], macroalgae [96], unicellular algae [97], estuarine copepod [98] and planktonic crustaceans [99]. Some authors confirm that water salinity influences the biodistribution and bioaccumulation of copper, affecting its toxicity [54,98,100–102]. Therefore, in oysters, copper accumulation was inversely related to salinity [100].

Copper causes toxicity by impairing the osmoregulation and ion regulation in the gill of numerous aquatic animals [54,55]. In brine shrimp, copper inhibited the Na/K ATPase and Mg^2+^ ATPase enzyme activity [52]. In mussel, *Mytilus galloprovincialis*, copper interfered with Ca^2+^ homeostasis in the gill, causing alterations in the Na/K ATPase and Ca^2+^ ATPase [53]. Copper depresses the transcription of key genes within the olfactory signal transduction pathway [103]. Additionally, copper toxicity can be induced by generating reactive oxygen species (ROS) [53,104].

It seems remarkable that phytoplankton species have different sensitivities to copper toxicity: resistant (diatoms), intermediate sensitivity (coccolithophores and dinoflagellates) and most sensitive (cyanobacteria) [105,106].

### 3.3. Dichlofluanid

Dichlofluanid (*N*-dichlorofluoromethylthio-*N*0-dimethyl-*N*-phenylsulphamide) has been commonly used as a herbicide on crops (Lee *et al.*, 2010). Dichlofluanid has a lower toxicity compared with other AF agents, although some studies have identified its toxic effects [107–109], such as embryotoxicity in sea urchin, *Glyptocidaris crenularis* [56].

### 3.4. DCOIT (Sea Nine 211^®^)

One of the new alternative biocides is 4,5-dichloro-2-*n*-octyl-4-isothiazolin-3-one (DCOIT), the active ingredient of the Sea Nine 211^®^ AF Agent manufactured by Rohm and Haas Company [110]. Aquatic microcosm and marine sediment studies demonstrate that the predominant route of DCOIT dissipation in the marine environment is its rapid biodegradation [110]. DCOIT predominantly undergoes biotic degradation under both aerobic and anaerobic conditions with biological degradation over 200 times faster than hydrolysis or photolysis [4,58,111]. Biodegradation is a very effective mechanism for the detoxification of the compound since the resulting metabolites are five orders of magnitude less toxic than the parent compound [112,113]. However, Sea-Nine antifoulant is acutely toxic to a wide range of aquatic organisms although no chronic toxicological effects have been observed in the extensive toxicology tests conducted on it [114]. DCOIT has a log K_OW_ of 2.8 and an aqueous solubility of 14 mg L^−1^ [4].

There are numerous studies that have investigated the toxicity and effects of DCOIT on marine animals. These studies demonstrated the following: larval mortality in crustaceans [57,58]: embryo-larva immobility and embryotoxicity in molluscs [46,47], embryotoxicity in echinoderms [59], embryotoxicity and inhibition of larval settlement in tunicates [47] and mortality in teleosts [46,115].

### 3.5. Diuron

Diuron (1-(3,4-dichlorophenyl)-3,3-dimethylurea) also persists in seawater, but it is less persistent in marine sediments with a half-life of 14 days [116,117]. Diuron is relatively soluble in water (35 mg L^−1^) and has a reported log *K*_OW_ of 2.8 [4]. Diuron is present at high concentrations in marine surface waters but it has only been detected at low concentrations in sediments [118,119]. Diuron is persistent in the marine environment and partitions poorly between water and sediments. It can remain suspended and available for uptake by marine organisms [120].

While the toxic effect of the antifoulant herbicide diuron to the photosynthetic aquatic biota has been widely studied, its sublethal effects on the different life stages of fish have been under-reported [121]. Diuron has been proven to be very toxic for the reproduction of the green freshwater alga *Scenedesmus vacuolatus* [60]. It has also been proven to affect planktonic and periphytic microalgae by reducing the chlorophyll *a* levels [61–63]. Moreover, it has been proven to be toxic to certain bacterial species [122–124].

### 3.6. Irgarol-1051^®^

Irgarol-1051 (2-methylthio-4-terbutylamino-6-cyclopropylamino-s-triazine) is a slightly soluble and moderately lipophilic triazine herbicide used in concert with copper to control fouling on boat hulls [125]. Irgarol inhibits electron transport in the photosystem II (PSII) [126] by binding to the D1 protein [127]. Irgarol may affect non-target photosynthetic organisms, such as phytoplankton, periphyton and aquatic macrophytes [128] when leaching into the marine environment [129].

Only a few studies have addressed the possible effect of Irgarol on marine non-target algae [130]. The effect of Irgarol on green alga *Dunaliella tertiolecta* [65], *Synechococcus sp* and *Emiliania huxleyi* [66], natural phytoplankton communities [131], periphyton colonization [129] and phytoplankton species [130,132,133] has been investigated and the results showed a decrease in growth, inhibition in cell number and a decrease in the photosynthetic activity of these organisms. These effects have been seen in many different marine plants and algae, such as the eelgrass *Zostera marina* [38,67], the brown macroalga *Fucus serratus* [69], the green macroalga *Enteromorpha intestinalis* [70] and the green macroalga *Ulva intestinalis* [71].

### 3.7. TCMS Pyridine

TCMS (2,3,5,6-tetrachloro-4-methylsulphonyl pyridine), which was used in both the textile and leather industries, is one of the more recent AF compounds introduced to the market [134]. The toxicity of TCMS towards living organisms has already been evidenced [29,135,136] and substantiated in *in vitro* studies [137,138]. TCMS has been found to cause immunotoxic effects at concentrations higher than 10 μM in haemocyte cultures of the colonial ascidian *Botryllus schlosseri*, causing oxidative stress in the process [71,72].

Both diuron and TCMS pyridine exerted immunosuppressant effects on the Botryllus hemocytes when used at concentrations higher than 250 μM and 10 μM, respectively, causing (i) deep changes in the cytoskeleton that irreversibly affect cell morphology and phagocytosis; (ii) induction of DNA damage; and (iii) leakage of oxidative and hydrolytic enzymes due to membrane alteration. Unlike organotin compounds, diuron and TCMS pyridine do not inhibit cytochrome-c-oxidase and only TCMS pyridine triggers oxidative stress.

### 3.8. Zinc Pyrithione

Zinc pyrithione (ZnPT) (bis(1hydroxy-2(1*H*)-pyridethionato-o,s)-(T-4)zinc), one of the most popular surrogate AF biocides, has long been widely used as algaecide, bactericide and fungicide [5,139]. ZnPT was found to be highly toxic to aquatic plants and animals [140], but it was assumed to be environmentally neutral because it could easily photo-degrade to less toxic compounds [140,141]. ZnPT is toxic to Japanese medaka fish (*Oryzias latipes*) and also causes teratogenic effects, such as spinal cord deformities in embryos and on the larvae of zebra fish (*Danio rerio*) [74] at very low sublethal concentrations [73]. However, there is a lack of data on the toxicity of ZnPT [139].

### 3.9. Zineb

Zineb (zinc ethylenebis-(dithiocarbamate)) is a widely used foliar fungicide with prime agricultural and industrial applications [142]. Zineb has been registered for use on fruits, vegetables, field crops, ornamental plants and for the treatment of many seeds [142]. It has also been registered as a fungicide in paints and for mould control on fabrics, leather, linen, painted and wood surfaces, and so on [143]. The occurrence of the dithiocarbamates in coastal environments was not reported until 2009 [144] although it is known that these compounds exhibit teratogenicity in fish embryos at relatively low concentrations [75].

### 3.10. Capsaicin, Econea and Medetomidine

Capsaicin, Econea and medetomidine can be collectively termed as “emerging” biocides [4]. Capsaicin (8-methyl-*n*-vanillyl-6-nonenamide) is a compound that may emerge as an AF biocide in the future. It has even been evaluated as a marine AF [4,145]. Econea (2-(p-chlorophenyl)-3-cyano-4- bromo-5-trifluoromethyl pyrrole) is being marketed as a metal-free biocidal additive replacement for copper [4]. Medetomidine (4-[1-(2,3-dimethylphenyl)ethyl]-3Himidazole), on the other hand, is a neuroactive catemine that has been shown to be effective in preventing barnacle cyprid settlement by interfering with the regulation of cement production [4,146].

## 4. Bioaccumulation

The bioconcentration of pesticides and other chemicals into aquatic organisms mainly proceeds by passive diffusion through gills, epithelial tissues, or the gastrointestinal tract [147]. Bioconcentration factors (BCFs) are available for certain biocides in specific tissues. They represent the concentration of a biocide in the tissue per concentration of the biocide in water (L kg^−1^) [4]. DCOIT has been shown to bioaccumulate in fish at very low levels following exposure to radiolabelled DCOIT [58]. There are no reports of the bioaccumulation of diuron with BCFs of 75 and 22 L kg^−1^, suggesting that its accumulation in aquatic organisms is unlikely [4,148]. Irgarol 1051 accumulates in freshwater macrophytes [149] and marine macrophytes [67] with BCFs of up to 30,000 L kg^−1^. It also accumulates in the green alga *Tetraselmis suecica* under laboratory conditions with BCFs of up to 150,000 mL g^−1^ [150]. In addition, the accumulation of Zineb in trout (*Salmo gairdneri*) is reported to be low with a BCF of <100 L kg^−1^ [4].

## 5. Resistance

Scientific evidence from bacteriological, biochemical and genetic data indicate that the use of active molecules in the biocidal products may contribute to the increased occurrence of antibiotic resistant bacteria. The selective stress exerted by biocides may favour the existence of bacteria expressing resistance mechanisms and their dissemination. Some biocides have the capacity to maintain the presence of mobile genetic elements that carry genes involved in cross-resistance between biocides and antibiotics. The dissemination of these mobile elements, their genetic organisation and the formation of biofilms, provide conditions that could create a potential risk of development of cross-resistance between antibiotics and biocides [151].

## 6. Conclusions

Biocides are used as components in paints to coat the structures of vessels, as a means of disinfecting aquaculture facilities and cages, as well as in controlling the biofouling phenomenon (antifouling). The use of biocides is not as well-regulated as drug use in aquaculture because the information available on the effects of these agents to the marine ecosystems is still limited. Hence, it is important to know the risks associated with the existence of those biocides in the marine environment. It is also important to evaluate the effects of these compounds through the continuous monitoring of biocide concentration profiles in water, sediment and biota to provide information that could lead to concerted action to ban or regulate their use.

## Figures and Tables

**Table 1:** The main antifouling biocides used in aquaculture and their effect on aquatic organisms.

Common Names a	Application a	Mode of Action a	Species	Effects
Chorothalonil	Fungicide	Inhibition of mitochondrial electron transport	**Crustaceans**	
	
				Behaviour
			*Cancer magister*	Larval mortality [43]
			*Penaeus duoramun*	Mortality [44]
			**Molluscs**	
	
			*Crassostrea virginica*	Growth [45]
				Embryotoxicity [46]
			*Mytilus edulis*	Embryotoxicity [47]
				Mortality [48]
			**Tunicates**	
	
				Embryotoxicity
			*Ciona intestinalis*	Inhibition of larval settlement [47]
			**Teleosts**	
	
			*Anguilla japonica*	Mortality [49]
			*Cyprinodon variegates*	Mortality [45]
			*Galaxias auratus*	Mortality [50]
			*Galaxias maculates*	Mortality [50]
			*Galaxias truttaceus*	Mortality [50]
			*Leiostomus xanthurus*	Mortality [44]
			*Pseudaphritis urvillii*	Juvenile mortality [51]

Copper pyrithione (CuPT)	Microbicide	Multi-site inhibitor (metabolic processes)	**Crustaceans**	
	
			*Artemia salina*	Inhibition of Na/K ATPase and Mg^2+^ ATPase enzyme activities [52]
			**Molluscs**	
	
			*Mytilus galloprovincialis*	Alterations in Na/K ATPase and Ca^2+^ ATPase activities [53]
			**Teleosts**	
	
			*Fundulus heteroclitus*	Alterations in gill osmoregulation [54]
			*Oncorhynchus kisutch*	Alterations in gill osmoregulation [55]

Dichlofuanid	Fungicide	Inhibitor of PS II electron transport	**Echinoderms**	
	
			*Glyptocidaris crenularis*	Embryotoxicity [56]

DCOIT (Sea-Nine 211^®^)	Herbicide	Inhibitor of PS II electron transport	**Crustaceans**	
	
			*Balanus amphitrite*	Larva mortality [57,58]
			**Molluscs**	
	
			*Crassostrea virginica*	Embryo-larva immobility [46]
			*Mytilus edulis*	Embryo-larva immobility and embryotoxicity [46,47]
			**Echinoderms**	
	
			*Hemicentrotus pulcherrimus*	Embryotoxicity [59]
			*Anthocidaris crassispina*	Embryotoxicity [59]
			**Tunicates**	
	
			*Ciona intestinalis*	Embryotoxicity and inhibition of larval settlement [47]
			**Teleosts**	
	
			Cyprinodon variegatus	Mortality [46]

Diuron	Herbicide	Inhibitor of PS II electron transport	**Algae**	
	
			*Scenedesmus vacuolatus*	Toxic for the reproduction [60]
			**Microalgae**	
	
			planktonic periphytic	Reduction of chlorophyll *a* levels [61–63]
			Teleosts	
			*Carassius auratus*	Acetylcholinesterase inhibition [64]

Irgarol-1051	Herbicide	Inhibitor of PS II electron transport	**Algae**	
	
			*Dunaliella tertiolecta*	Decreasing in growth, inhibition of cell number and decrease in the photosynthetic activity [38,65–70]
			*Synechococcus sp*	
			*Emiliania huxleyi*	
			*Zostera marina*	
			*Fucus vesiculosus*	
			*Enteromorpha intestinalis*	
			*Ulva intestinalis*	

TCMS pyridine (2,3,3,6-tetrachloro-4- methylsulfonylpyridine)	Fungicide	Inhibitor of mitochondrial electron transport	**Tunicates**	
	
			*Botryllus schlosseri*	Immunotoxic [71,72]

Zinc pyrithione (ZnPT)	Microbicide	Multi-site inhibitor (metabolic processes)	**Teleosts**	
	
			*Oryzias latipes*	Embryotoxicity [73]
			*Danio* rerio	Embryo-larva [74]

Zineb	Fungicide	Multi-site inhibitor (metabolic processes)	**Teleosts**	
	
			*Salmo gairdneri*	Embryotoxicity [75]

a From [76].
